# Biocontrol potentiality and plant growth-promoting traits of endophytic *Enterobacter cloacae* RO2 isolated from *Portulaca oleracea* L. against *Bipolaris sorokiniana*

**DOI:** 10.1038/s41598-026-54498-w

**Published:** 2026-06-04

**Authors:** Asmaa H. Mohamed, Rabab A. Metwally, Asmaa S. Taha, Shereen A. Soliman

**Affiliations:** Botany and Microbiology Department, Faculty of Science, Zagazig University, Zagazig, 44519 Egypt

**Keywords:** Biocontrol agents, Endophytes, *Enterobacter cloacae*, Plant growth promoting bacteria, Seed biopriming, Biofertilizers, Biotechnology, Microbiology, Plant sciences

## Abstract

In agricultural applications, plant growth-promoting bacteria (PGPB) have demonstrated their efficacy as both biofertilizers and biocontrol agents against several phytopathogens. This study reports, for the first time, the isolation of an endophytic *Enterobacter cloacae* from the medical plant *Portulaca oleracea* L., which produces iturin-like lipopeptides effective against *B. Sorokiniana* that causes severe diseases that affects several important crops. The present study investigates the ability of *E. cloacae* as a PGPB and a producer of bioactive metabolites that participate in the biological control of *Bipolaris sorokiniana*. Twelve endophytic bacterial isolates obtained from *P. oleracea*, grown in El-Sharkia Governorate, Egypt, were examined for their antagonistic activity against *B. sorokiniana* through the disk diffusion method. Also, the most active isolates (2, 5, 7 and 8) were investigated using a dual culture technique against *B. sorokiniana in vitro*, and the percentages of inhibition were 43.48, 40.55, 36.60, and 35.57%, respectively. Therefore, the most active endophytic bacterial isolate (2) was molecularly identified as *E. cloacae* strain RO2 with accession number PV739238. *In vitro* assessment of PGP traits of *E. cloacae* strain RO2 proved that it can produce protease, amylase, cellulase, in addition to nitrogen fixation, siderophore production, phosphate and calcium carbonate solubilization and ammonia production. *E. cloacae* caused irregular mycelial growth and morphological deformation in *B. sorokiniana* hypha. HPLC analysis of *E. cloacae’*s lipopeptide extract revealed the occurrence of lipopeptide like Iturin that had inhibitory effects on *B. sorokiniana*. In addition, the growth-promoting potential of *E. cloacae* was evaluated on wheat (*Triticum aestivum* L.) seeds bio-primed with it, and the results showed enhanced shoot height, radicle length, fresh and dry weight of seedling as compared to the control. Consequently, *E. cloacae* strain could be used as biofertilizers and biocontrol agent in agriculture to reduce the risk of chemical pesticides.

## Introduction

*Bipolaris sorokiniana*, a major fungal pathogen that mostly affects wheat in warmer regions^1^, it is the causal agent for seedling blight, head blight, foliar blight (spot blotch), black point, and common root rot spot blotch disease, resulting in considerable yield losses up to 88%^2–5^. *B. sorokiniana*, classified as a hemibiotrophic fungus, initially draws nutrition from living host cells during infection^6^. Chemical fungicides are one traditional controlling technique of fungal pathogens that exhibit powerful antifungal characteristics against the majority of plant diseases^7^. These chemical substances are expensive, with limited efficacy, and have a detrimental impact on human health and non-target species in the environment^8^. The prolonged application of these chemical compounds has led to the development of resistant pathogens and the precipitation of fungicide residues in the soil, posing dangerous threats to the ecosystem^9^. An effective and environmentally friendly substitute for the use of these chemical fungicides is biological control, especially when there is a need for environmental protection toward sustainable farming practices^10^. Researchers are now paying more attention to effective biocontrol techniques^11^.

Biological control agents include beneficial microbes, which prevent plant diseases and enhance immunity^12^. They act as an ecologically secure and economically viable method for controlling pathogens. *Portulaca oleracea* L. that has garnered increasing scientific attention as a multifunctional plant with nutritional and pharmacological significance^13–16^. This plant is widely recognized as one of the richest sources of omega-3 fatty acids, flavonoids, alkaloids, terpenoids, vitamins^17^ and essential minerals, all of which contribute to its strong antioxidant capacity^18^. These compounds play a crucial role in scavenging reactive oxygen species (ROS) and mitigating oxidative stress. *P. oleracea* is a resilient plant capable of growing under diverse environmental conditions. In Egypt, *P. oleracea* is commonly known as (Rejlah). In addition to its phytochemical importance, *P. oleracea* contains a variety of beneficial bacterial endophytes that colonize its internal tissues without posing a threat^19^. Through processes like phytohormone production, nitrogen fixation, phosphate solubilization, and induction of systemic resistance, these endophytic bacteria which frequently belong to genera like *Bacillus*,* Pseudomonas*, and *Enterobacter* play crucial roles in fostering plant growth and improving stress tolerance^20–22^. Further supporting the plant’s therapeutic usefulness, a number of *P. oleracea* isolates have shown the capacity to generate secondary metabolites with antibacterial and antioxidant properties^16–19^.Therefore, *P. oleracea’s* symbiotic relationship with its endophytic microbiota highlights its dual significance as a medicinal species and a potential source of bioactive and PGP microbes.

Moreover, bacterial endophytes are able to improve plants’ immune responses and, by forming a symbiotic relationship, they have the ability to suppress plant pathogenic fungi while simultaneously encouraging plant growth^23,24^. Endophytic bacteria can suppress pathogens by producing lytic enzymes and secondary metabolites, occupying plant tissue space, and improving plant defenses^25^. These bacteria’s biocontrol strategies can be divided into two distinct groups: direct and indirect. The direct mechanism involves antibiosis, parasitism, competition, and the production of extracellular enzymes. They produce antifungal and antibacterial secondary metabolites, which prevent the growth of phytopathogenic microorganisms^26^, including iturin, surfactin, fengycin, polymyxin, phenazine-1-carboxylic acid, and pyrrolnitrine, which are well known for their antimicrobial activity^23,27^. Hydrolytic enzymes, including chitinase, cellulase, β-1,3-glucanase, and protease, produced by endophytic bacteria that may attack the pathogen cell wall, have been extensively documented and may digest different polymers, such as lipids, proteins, chitin, and cellulose^28,29^. Plant pathogens may also be suppressed by endophytic bacteria through competition for space and nutrients. Also, siderophores produced by endophytes have exhibited biocontrol activity^30^. In contrast, the indirect bio-control strategy involves the induction of plant immunity^31^.

*Enterobacter* are frequently described as endophytic diazotrophs in many plants^32^. Remarkably, *E. cloacae* was mentioned as a possible biological control agent against number of plant pathogens that release volatile organic compounds (VOCs) which alter genes involved in plant defense^33–35^. *Enterobacter cloacae* enable wheat plants to grow and become more resilient to stress^36,37^. *E. cloacae* BHUAS1 and *Enterobacter* E1S2 had notable PGP traits and were able to produce soluble phosphate, Indole-3-acetic acid (IAA), siderophore, ammonia, HCN and showed zinc solubilization^38^. Furthermore, *E. cloacae* considerably decreased disease incidence by 63%, suggesting that it could be employed as a biocontrol agent to suppress damping-off disease caused by *Pythium aphanidermatum* in cucumber^39^, that was attributed to pathogen suppression and induction of systemic resistance that was indicated by a notable increase of salicylic acid as well as total phenols^20,34^.

Inoculation of wheat with endophytic *Bacillus* spp. and *Enterobacter* spp. has been shown to increase biomass and grain yield under controlled conditions, while maize and rice inoculated with *P. fluorescens* and *K. pneumoniae* exhibited improved drought and salinity tolerance through enhanced antioxidant activity and osmolyte accumulation^40^. Despite the abundance of research in the field of using endophytes as biological control agents, however, endophytic *E. cloacae* from medicinal plants such as *P. oleracea* remain poorly explored. The objectives of the current work was to screen and evaluate novel endophytic bacterial isolates for their capacity to inhibit *B. sorokiniana*, and this encourages a decrease in the usage of chemical pesticides. Additionally, the plant growth-promoting properties of the most active isolate were evaluated.

## Materials and methods

### Plant sample collection, and endophyte isolation

Plant samples were randomly gathered from fields in Minia Al-Qamh, El-Sharkia Governorate, Egypt, (30°30’16.6"N 31°21’06.8"E). Endophytes were isolated from leaves and stems of healthy *Portulaca oleracea* L. plant segments (2 cm in length) following the method of Huang et al.^41^. Plant samples of *Portulaca oleracea* L. were collected after obtaining the required permission from the relevant authority. The plant material was authenticated by Dr. Samir S. Teleb, Assistant professor of Taxonomy, Department of Botany and Microbiology, Faculty of Science, Zagazig University, Egypt. A voucher specimen was deposited in HEU Herbarium , Botany and Microbiology Department, Faculty of Science, Capital University (formerly Helwan University), Cairo, Egypt, under voucher number [0111107], to ensure future reference and public accessibility of the plant material used in this study. Plant samples were rinsed with tap water, and then sterilized with 70% (v ⁄ v) ethanol for 2 min. The segments were then submerged in 10% NaOCl for 10 min, washed with sterile water three times and then dried on a sterilized filter paper. After surface sterilization, segments were inserted on potato dextrose agar (PDA) medium and incubated at 30 °C for 2–3 days until bacterial growth appeared. Different colonies were aseptically inoculated into fresh PDA until pure cultures were obtained.

### Pathogenic fungus

The current study used *Bipolaris sorokiniana* OP714480 fungus that was previously isolated from diseased leaves of wheat and barley plants and its ability to cause spot blotch disease on barley was studied^42^.

### Initial assessment of the antagonistic features of the isolated endophytic bacteria

Petri dishes containing 20 mL of PDA were supplied with 1 mL of *B. sorokiniana* spore suspension. Plates were allowed to solidify then inoculated with bacterial discs (10 mm) from 48-h-old cultures of twelve endophytic bacterial isolates. The plates were left in a refrigerator for 2 h and then incubated for 5 days at 28 °C. At the end of the incubation period, the inhibition zones were determined^43^. The most active isolates were chosen for further studies.

### *In vitro* antifungal activity of the most active isolates against *Bipolaris sorokiniana*

The most bioactive endophytic bacterial isolates were investigated for their antifungal potential activity against *B. sorokiniana* using the dual culture technique. A fungal disc (10 mm) of a five-day culture was inoculated on one side of the petri plates. On the other hand, the isolates were streaked on the opposite side. Plates with only *B. sorokiniana* disc were regarded as the controls^44^. After five days of incubation at 28 °C, the inhibitory zone confirmed the antagonistic properties of the endophytic bacteria. To ensure accuracy, the experiment was performed twice and four replicates were measured for each isolate. The radial growth inhibition percentage in comparison to the control was estimated using the following equation^45^.

$$\text{Percent of Inhibition}\:\% \left(\mathrm{I}\right)=\left[\left(\mathrm{C}-\mathrm{T}\right)/\mathrm{C}\right] \times 100.$$

*C is the radial growth of the control and T is the radial growth of the treatment.

### Morphological and molecular Identification of the most active isolate

The selected isolate for its antifungal activity was cultivated on nutrient agar for 24 h. Colonies’ growth pattern and Gram staining were used for morphological identification according to standard microbiological techniques^46^. Concerning molecular analysis, the isolate was grown on liquid nutrient media at 30 °C, for 24 h, and 180 rpm. The culture was then centrifuged at 6,000 ×g for 10 min. The cell pellet was used for genomic DNA extraction using a standard method^47^ while supernatant was discarded. PCR was used to amplify the 16 S rRNA gene using the universal primers (27 F) AGAGTTTGATCMTGGCTCAG and (1492R) TACGGYTACCTTGTTACGACTT according to^48^. Sequences were BLAST searched against homologous bacterial 16 S ribosomal RNA sequences using NCBI (http://blast.ncbi.nlm.nih.gov/). A phylogenetic tree was constructed based on the MEGA 7 software^49^. The sequence was then deposited in GenBank, and an accession number was assigned.

### Growth enhancement traits of the most active isolate

#### Siderophore detection

The siderophore production of the most active isolate was evaluated qualitatively on iron-free S7 agar minimal medium. Plates were inoculated with a 24 h-old bacterial culture^50^. After 5 days of growth at 30 °C, 10 ml of Chrome Azurol S (CAS) agar was put on top of agar plates that contained the chosen isolate. The formation of yellow-orange halo zone around bacterial growth noticed after 1 h of incubation, considered as positive sign for siderophore production.

#### Nitrogen fixation

To examine the ability of the selected isolate to fix nitrogen, the bacterial isolate was cultivated on nitrogen-deficient medium^51^. Jensen’s medium plates are composed of 20 g sucrose, 1.0 g K_2_HPO_4_, MgSO_4_·7H_2_O at 0.5 g, NaCl at 0.5 g, FeSO_4_·7H_2_O at 0.1 g, CaCO_3_ at 2.0 g, Na_2_MoO_4_ at 0.005 g and agar at 15.0 g per liter of sterile distilled water. Plates were incubated at 30 ± 2 °C for 7 days. The bacterial growth on plates was considered as positive results for nitrogen-fixation.

#### Phosphate solubilization

Qualitative phosphate solubilization ability of the selected isolate was evaluated^52^. Using Pikovskaya agar medium (glucose 10 g L^–1^, Ca_3_(PO_4_)_2_ 5 g L^–1^, (NH_4_)_2_SO_4_ 0.5 g L^–1^, NaCl 0.2 g L^–1^, MgSO_4_·7H_2_O 0.1 g L^–1^, KCl 0.2 g L–1, FeSO_4_·7H_2_O 0.002 g L^–1^, yeast extract 0.5 g L^–1^, MnSO_4_·2H_2_O 0.002 g L^–1^, and agar 15 g L^–1^). After ten days of incubation at 30 °C, the potentially of the isolate to use inorganic phosphate Ca_3_(PO_4_)_2_ was assessed by the development of a transparent zone surrounding the colony.

#### Detection of ammonia

Qualitatively, the bacterial isolate was tested for ammonia production. Test tube contain 10 mL peptone water was inoculated and incubated for 48 h at 32 ± 2 °C. After that, Nessler’s reagent (0.5 mL) was added. In a positive ammonia production test, brown to yellow coloration was obtained^53^.

#### Hydrolytic enzyme production

The production of hydrolytic enzymes, such as protease, amylase, and cellulase was verified for the selected bacterial isolate. Substrate agar media without bacterial inoculation used as negative controls.

##### Protease production

The Skim Milk Agar medium was used for assessing protease activity. Bacteria incubated for 24 h at 32 °C. Clear proteolytic zone surrounding the colony was considered as a positive result for enzyme production^54^.

##### Amylase production

To detect starch hydrolysis activity of the bacterial isolate, starch agar plates were cultivated and incubated for 24 h at 32 °C, the plates were rinsed with Iodine. A colorless zone around colonies indicating the hydrolysis of starch^55^.

##### Celulase production

Carboxymethylcellulose (CMC) agar medium was used to determine cellulase activity. The plates were inoculated and incubated at 32 °C for two days. The hydrolysis zones were visualized using 0.1% Congo Red for 20 min and then destained with 1 M sodium chloride^56^.

### Morphological deformation of *B. sorokiniana* mycelium due to the antifungal activity of the selected endophytic bacterial isolate

The deformation of the mycelium of the pathogenic fungus (*B. sorokiniana*) caused by the most bioactive endophytic bacterial isolate was studied on PDA plates. The hyphal filaments from the front lines were examined under a light microscope (Leitz WETZLAR, Wetzlar, Germany) for deviations^57^ in contrast with the control plates.

### Extraction and partial purification of antifungal lipopeptide metabolite of the selected endophytic bacterial isolate

The most active isolate was cultured using 300 mL of LB medium (pH 7), in 500 mL conical flasks for 72 h, at 30 °C and 160 rpm. After incubation, the broth culture was centrifuged for 15 min at 10,000 rpm and 4 °C to obtain the cell-free supernatant. The pH was adjusted to pH 2.0 with 6 M HCl overnight. The precipitate was obtained by centrifugation (4 °C, 6000 rpm, and 15 min) and then methanol was used for extraction. Bioassay of the antifungal lipopeptide of the crude extract was conducted using the well diffusion method (100 µL) against *B. sorokiniana* on PDA. The crude extract was re-dissolved in 1 mL of 70% methanol^58^. Then, 500 µL of dissolved extract was filtered through a 0.2 μm syringe filter before analyzed by reversed-phase high-performance liquid chromatography (RP-HPLC). Instrument LC1620A Liquid Chromatograph equipped with a C18 solid-phase extraction column (250 mm × 4.6 mm, 5 μm), with a column temperature of 25 °C. The mixture of methanol and water in a ratio of 70:30 (v/v) was used as a mobile phase with a UV-VIS wavelength detector. The flow rate was set to 1.0 mL/min. An injection volume of 20 µL of sample and standard Iturin A (Sigma - USA) were injected under standardized conditions. Detection was carried out by measurement of the absorption at 240 nm, comparing the area under peak of the lipopeptide sample with that of the reference lipopeptide.

### Bio-priming of wheat grains with the selected endophytic bacterial isolate

The PGP ability of the selected bacterial isolate was investigated using the germination assay test under sterilized conditions. The grains of wheat (*Triticum aestivum* L.) cultivar (Sakha 93) were obtained from the Field Crop Research Institute, Agricultural Research Center, Giza, Egypt. Grains were surface sterilized with 2% (w/v) NaOCl for 5 min and washed with sterile water. Seeds were immersed in a 10 mL bacterial suspension 10^9^(CFU/mL) and soaked in 10 mL sterile water as a control for two days under sterilized conditions to avoid contamination until the emergence of the radicle^59^. Each treatment was replicated three times (3 × 10 grains). Grains with a uniform shape and size were incubated under aseptic conditions at 22 °C with a 16/8 h day/night on water agar media with streptomycin (15 mg L^− 1^). Seedling’s growth-related parameters, including root and shoot length, fresh and dry weight, were determined separately, 4 days after sowing for both treatments.

### Statistical analysis

Prior to analysis, data were tested for normality using Levene’s test. The results are the mean ± standard error. The data were statistically analyzed using SPSS (Version 16.0, SPSS Inc., Chicago, IL, USA). One-way ANOVA followed by Duncan’s multiple range test (DMRT, *p* ≤ 0.05) Duncan^60^ was used in Table 2 to compare means. While data in Table 3, independent samples Student’s t-test was used supported by corresponding t-values, degrees of freedom (df), and exact p-values which have been added to the results section.

## Results and discussion

**Fig. 1:**
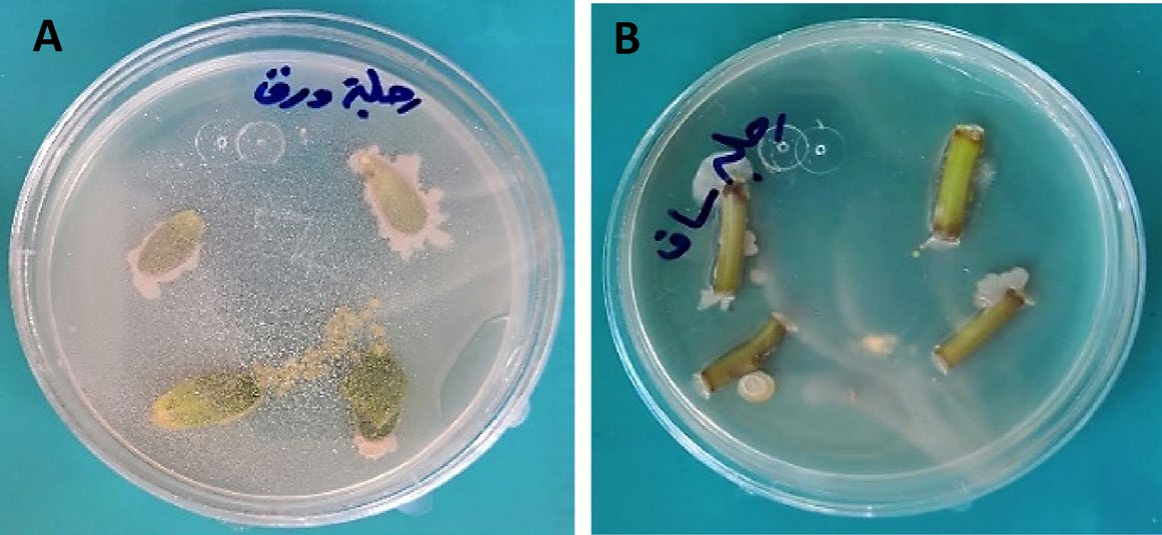
Endophytic bacterial isolation from the leaves (**A**) and (**B**) stems of *Portulaca oleracea* L.

**Fig. 2:**
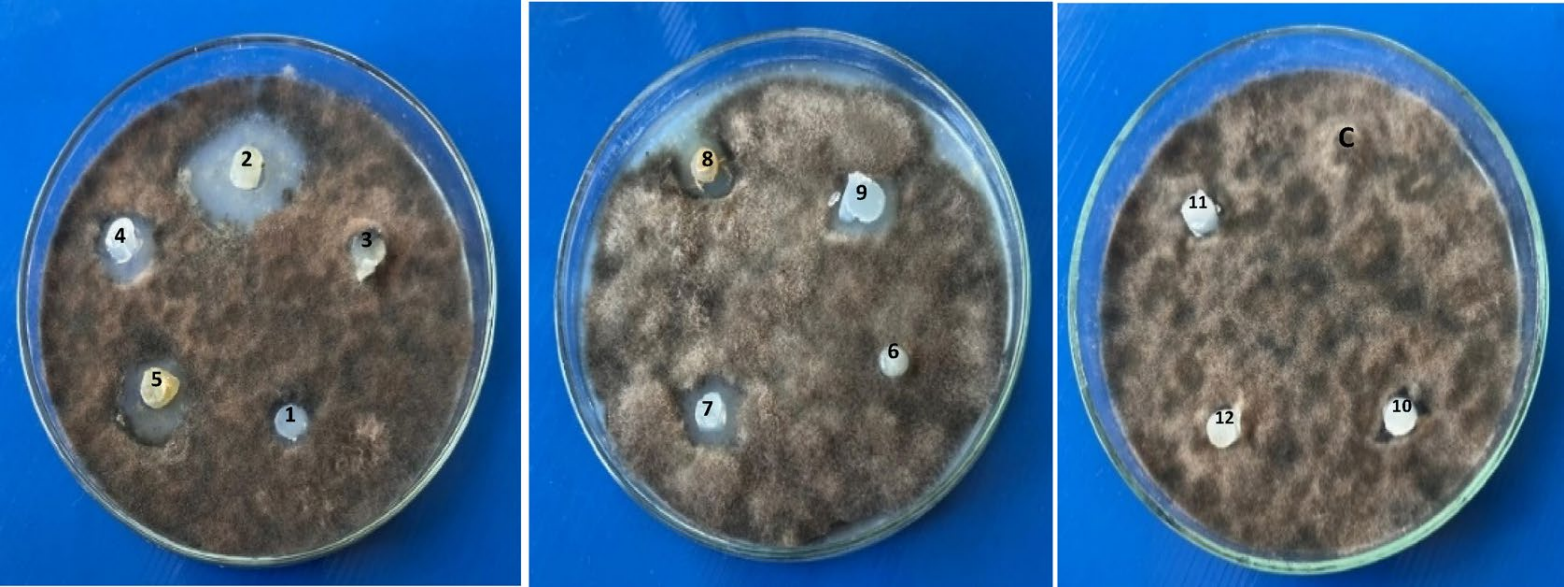
Primary screening of the antagonistic activity of the twelve endophytic bacterial isolates evaluated against *Bipolaris sorokiniana* through disk diffusion method.

An eco-friendly approach for protecting crops from phytopathogenic fungi is the use of biological control agents, such as PGPBs, which are effective in restricting disease and promoting plant growth, making them a better choice for bio-preparations^20^. Hallmann et al.^61^ stated that the most common bacterial endophytes collected from plant tissues were *Agrobacterium*,* Pseudomonas*,* Enterobacter*, and *Bacillus*. Endophytic bacteria could be an efficient and secure alternative strategy for the biocontrol of phytopathogenic fungi, improve plant protection and boost plant immunity^62^. In the present work, twelve endophytic bacteria were isolated from leaves and stems of *P. oleracea* plants, as shown in Table 1 and Fig. 1. These isolates were examined to evaluate their antagonistic activity against *B. sorokiniana*, the most destructive wheat disease that causes significant yield loss^5,63^, through the disc diffusion technique (Fig. 2).

**Table 1:** Primary screening of the antagonistic activity of the twelve-isolated endophytic bacterial evaluated through disk diffusion method against *Bipolaris sorokiniana*.

Isolate No.	Antifungal activity	Isolate No.	Antifungal activity
1	-	7	++
2	++++	8	++
3	-	9	+
4	+	10	+
5	+++	11	-
6	-	12	-

Data are based on four replicates of each experiment. + represents < 10 mm wide zone; ++ represents < 15 mm wide zone; +++ represents > 15 mm wide zone and ++++ represents > 20 mm wide zone.

**Table 2:** *In vitro* antifungal activity of the most active isolates against *Bipolaris sorokiniana* in a dual culture assay after five days on PDA media.

Isolate No.	Radial growth of Bipolaris sorokiniana (cm)	Inhibition %
Control	6.833 ± 0.088a	-
2	3.867 ± 0.067c	43.48 ± 2.300a
5	4.067 ± 0.067c	40.55 ± 2.145ab
7	4.333 ± 0.088b	36.60 ± 1.936ab
8	4.400 ± 0.058b	35.57 ± 1.882b

*Data are the mean ± SE (standard error). Different letters indicate significant differences among treatments using a one-way ANOVA followed by the Duncan’s multiple range test (*p* < 0.05).

**Table 3:** Plant growth promotion parameters analyzed on *Triticum aestivum* after seed biopriming with H_2_O as control and Endophytic *Enterobacter cloacae* after 4 days of growth.

Parameters	Treatments	Mean ± SE	t-value	df	*p*-value	Significance
Shoot height (cm)	H_2_O primed (Control)	1.54 ± 0.082	-4.301	4	0.013	*
	*Enterobacter cloacae*-primed	2.14 ± 0.113				
Radicle length (cm)	H_2_O primed (Control)	3.60 ± 0.190	-3.911	4	0.017	*
	*Enterobacter cloacae*-primed	4.85 ± 0.257				
Seedling length (cm)	H_2_O primed (Control)	5.14 ± 0.272	-4.030	4	0.016	*
	*Enterobacter cloacae*-primed	6.99 ± 0.369				
Seedling FW (gm)	H_2_O primed (Control)	1.105 ± 0.058	-0.795	4	0.471	ns
	*Enterobacter cloacae*-primed	1.173 ± 0.062				
Seedling DW (gm)	H_2_O primed (Control)	0.449 ± 0.024	-0.554	4	0.609	ns
	*Enterobacter cloacae*-primed	0.468 ± 0.025				

*Data are presented as the mean ± SE (standard error). An independent samples Student’s t-test was used to compare means between the two groups. The results include the corresponding t-values, degrees of freedom (df), and exact p-values.

**Fig. 3:**
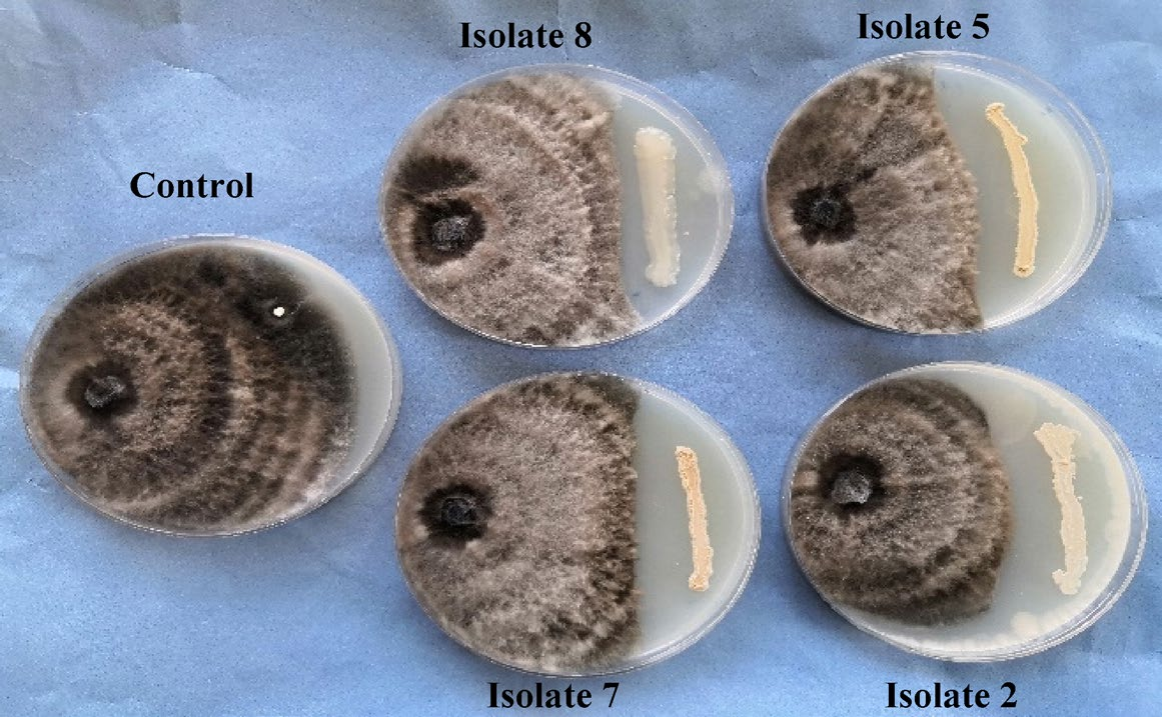
*In vitro* antifungal activity of the most active isolates against *Bipolaris sorokiniana* in a dual culture assay after five days on PDA media.

**Fig. 4:**
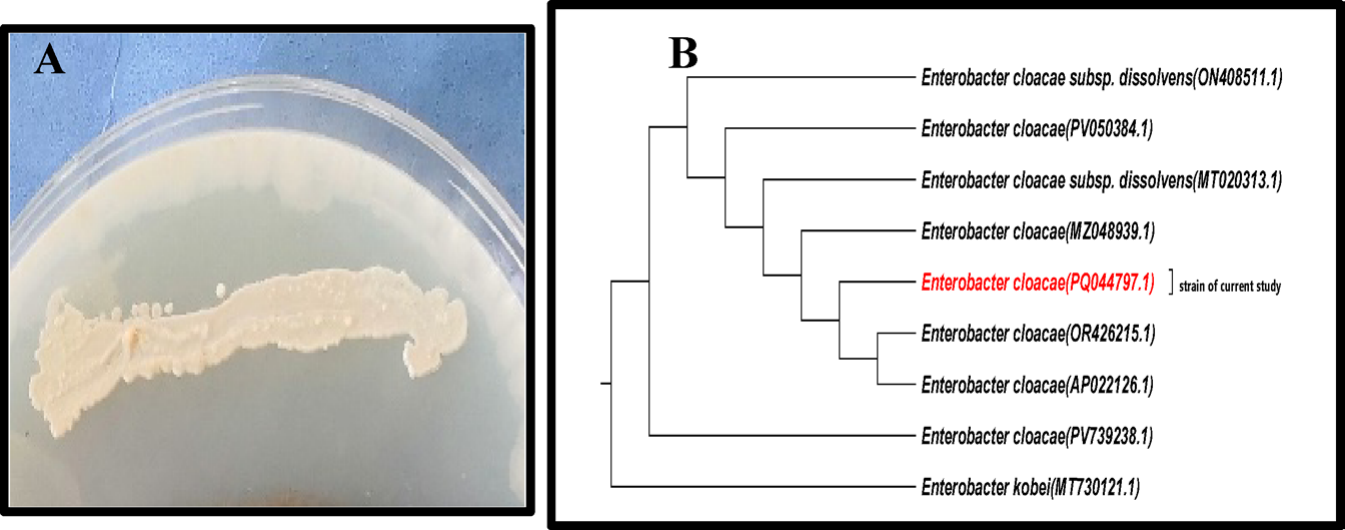
Morphological and molecular identification of the most active isolate (**A**) Colony morphology, and (**B**) Phylogenetic tree of *Enterobacter cloacae* based on 16 S rRNA sequences.

The disk diffusion method demonstrated the antagonistic activity of the endophytic bacteria in comparison to the control. The width of the inhibition zones was calculated as: + represents < 10 mm wide zone; ++ represents < 15 mm wide zone; +++ represents > 15 mm wide zone, and ++++ represents > 20 mm wide zone. The most active isolates against *B. sorokiniana* were isolates no. 2, 5, 7 and 8, as shown in Table 1, while isolates 4, 9 and 10 had moderate inhibition zones. In contrast, 1, 3, 6, 11, and 12 did not have any inhibitory effects on the pathogenic fungus. A variety of microbial antagonists have been shown to inhibit *B. sorokiniana* *in vitro* in a number of previous investigations^64,65^. For instance, *P. fluorescens* UBSPF-10^66^. *Pseudomonas* spp. and *Bacillus* spp. were also found to be effective in suppressing *B. sorokiniana* in wheat^67,68^.

Therefore, the most active endophytic bacterial isolates (2, 5, 7 and 8) were investigated for their potential antifungal activity against *B. sorokiniana in vitro* using a dual culture technique as shown in Fig. 3. The results showed that the inhibition in radial growth revealed the bacteria’s antagonistic activity, as shown in Table 2, the percentages of inhibition were 43.48, 40.55, 36.60, and 35.57% for 2, 5, 7 and 8, respectively. Likewise, *B. subtilis* TE3 can hamper the mycelial growth of *B. sorokiniana* by 55%^69^. Basak et al.^64^ stated that *P. fluorescens* (T36) and *B. amyloliquefaciens* (T35) showed 64.09% and 57.09% inhibition of *B*. *sorokiniana*, respectively.

According to the dual culture technique results, the most active bacterial isolate (2) was selected and identified morphologically using Gram stain; the isolate appeared as a Gram-negative and rod-shaped bacterium. In addition, the identification was confirmed using the 16 S rDNA gene sequence and the obtained partial sequence was deposited in the GenBank under accession number PV739238, as shown in Fig. 4. The isolate was identified as *Enterobacter cloacae* strain RO2. Among PGPB, the Gram-negative *Enterobacter* genus possesses PGP traits, including its ability to produce siderophores, solubilize phosphorus, fix N_2_, create antimicrobial compounds, and produce hydrolytic enzymes^20^. Many endophytic *Enterobacter* spp. have been isolated from different plants such as *E. cloacae* from citrus and maize plants^70^, *E. asburiae* from sweet potato^71^ and *E. sakazakii* and *E. agglomerans* from soybean^72^.

The investigated *E. cloacae* strain RO2 was isolated from a plant-associated environment, suggesting ecological specialization distinct from clinical isolates. The *E. cloacae* species is a diverse group of bacteria that has been found in various environments, ranging from plants to soil to humans. Plant-associated isolates contain genes involved in plant growth promotion and biocontrol activity (e.g., siderophore production, hydrolytic enzymes, and antimicrobial metabolites). Whereas the clinical strains are enriched in virulence factors and multidrug resistance genes. These differences suggest the ecological niche adaptation^73^. Shastry et al.^74^ concluded that the plant-associated *E. cloacae* strain exhibits genomic features consistent with an endophytic lifestyle, including genes involved in plant colonization, and nutrient acquisition, while lacking key virulence determinants associated with clinical isolates. The genome sequencing studies have demonstrated that clinical *E. cloacae* isolates exhibit distinct genomic characteristics, including virulence-associated genes^75^. Moreover, several studies have confirmed the safe application of environmental *E. cloacae* strains in agricultural systems, where they function as PGPR and biocontrol agents without exhibiting pathogenic traits under experimental conditions^76,77^.

**Fig. 5:**
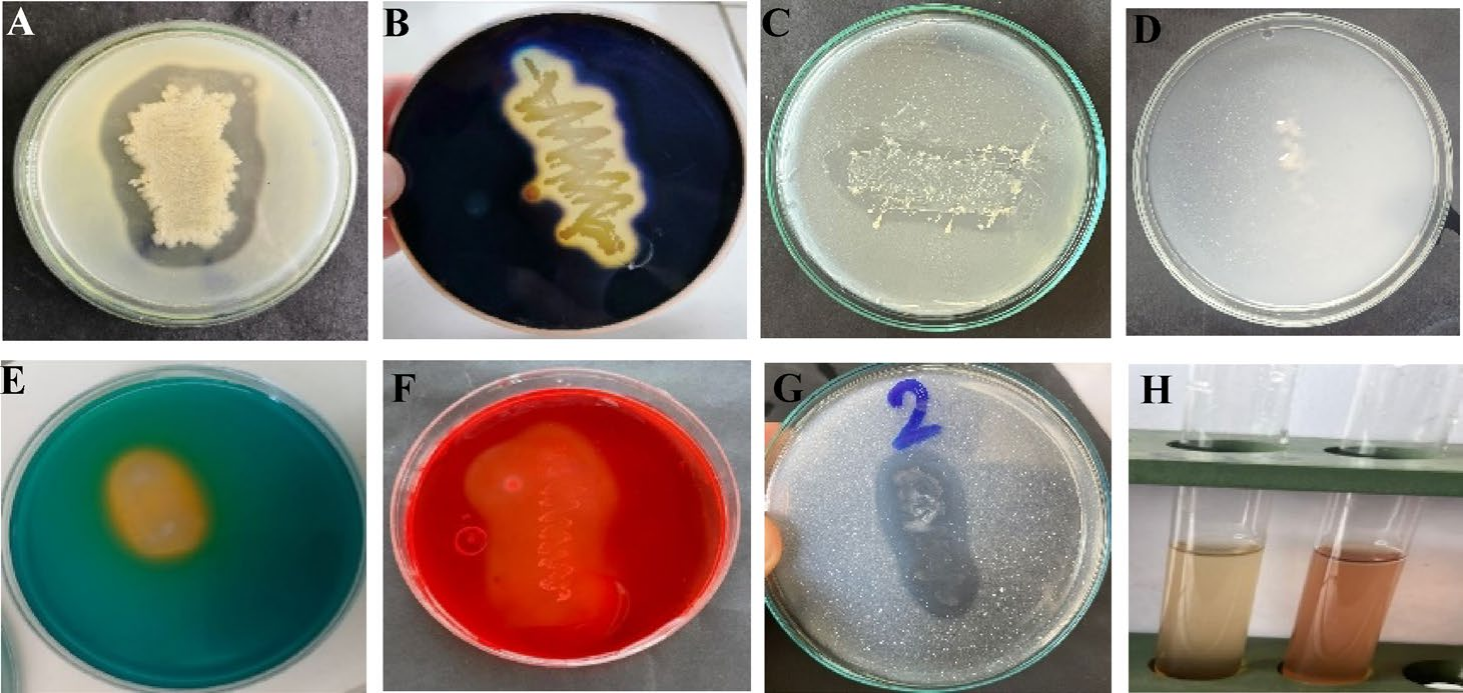
In-vitro assessment of plant growth promoting (PGP) traits of the most active isolate *Enterobacter cloacae* (**A**) Protease activity, (**B**) Amylase activity, (**C**) Phosphate solubilization, (**D**) Nitrogen fixation, (**E**) Siderophore production, (**F**) Cellulose activity, (**G**) calcium carbonate solubilization and (**H**) Ammonia production.

An *in vitro* assessment of the PGP traits of *E. cloacae* strain RO2 was evaluated as shown in Fig. (5A-H). A diverse extracellular enzymatic activity of *E. cloacae*, such as protease (Fig. 5A), amylase (Fig. 5B), and cellulose (Fig. 5F), was examined. Our result revealed that *E. cloacae* displayed a positive response to protease, amylase, and cellulase, which appeared as well-defined clearing zones. These positive results show that *E. cloacae* RO2 is a potent antagonist to suppress fungal pathogen growth because it degrades the main components of the fungal cell wall. Similarly, rhizobacterial isolate *Pseudomonas* sp. M2 produce hydrolytic enzymes (protease, cellulase, and chitinase) and siderophore^78^.

The results illustrated that *E. cloacae* produce yellow-orange zone on the CAS agar, confirming the siderophore-producing potential, which means it competes for Fe availability (Fig. 5E). Siderophore is an iron binding low molecular weight organic molecule that bacteria synthesize intracellularly, it increase availability of iron, stimulate plant growth, improving plant nutrition and protection against phytopathogens. Siderophore production is a microbial mechanism considered a PGP trait because it may be involved in biocontrol activity, increase the P availability, and consequently improve the yield of crop^38,79^. The similar study was also reported by Yue et al.^80^ under Fe limitation, siderophore-producing microorganisms, such as *Bacillus* sp. WR12, can be used as bio-fertilizers to enhance wheat growth by increasing Fe acquisition and alleviating growth. Macedo-Raygoza et al.^81^ observed siderophore production *in E. cloacae*. Similar study was also found in *Enterobacter* AABM_9’s siderophores significantly improved peanut seedling length compared to other legumes^82^. Thus, siderophore-producing bacteria can serve as bio-fertilizers to boost wheat growth and as biological control agents against plant pathogens^65^.

Our results showed a positive response of *E. cloacae* as it can grow on Jensen’s medium plates; this indicates *E. cloacae* as nitrogen fixers (Fig. 5D). Our results also showed that *E. cloacae* exhibited a positive response to ammonia (NH_3_, Fig. 5H) production and calcium carbonate solubilization **(**Fig. 5G). Similarly, *E. cloacae* promote the growth and health of banana crops^81^. Therefore, PGPR strains of *Enterobacter* nowadays are considered as bio-fertilizers and could mitigate the effects of climate change. Sood et al.^83^ and Jaggi et al.^84^ stated the positive reaction of siderophore and ammonia production, in addition to phosphate solubilization for some bacterial isolates.

Our result investigated that *E. cloacae* formed a clear zone on Pikovskaya media (Fig. 5C), confirming the potential phosphate solubilization activity. *E. cloacae* and *K. pneumoniae* have been previously reported to be phosphate solubilizers^85^. Khalifa et al.^86^ reported that endophytic *E. cloacae* MSR1 showed characteristics that would stimulate plant development and could be considered as biofertilizer. Our results are also consistent with Kumar and Prasad^38^, who proved that *E. cloacae* BHUAS1 exhibited significant PGP traits and has the ability to produce soluble phosphate, IAA, siderophore and ammonia under various treatments. Similar results showed that *Enterobacter* spp. can enhance plant growth in several ways, including phosphate solubilization^37,87^; nitrogen fixation^87^, and IAA production^88^.

**Fig. 6:**
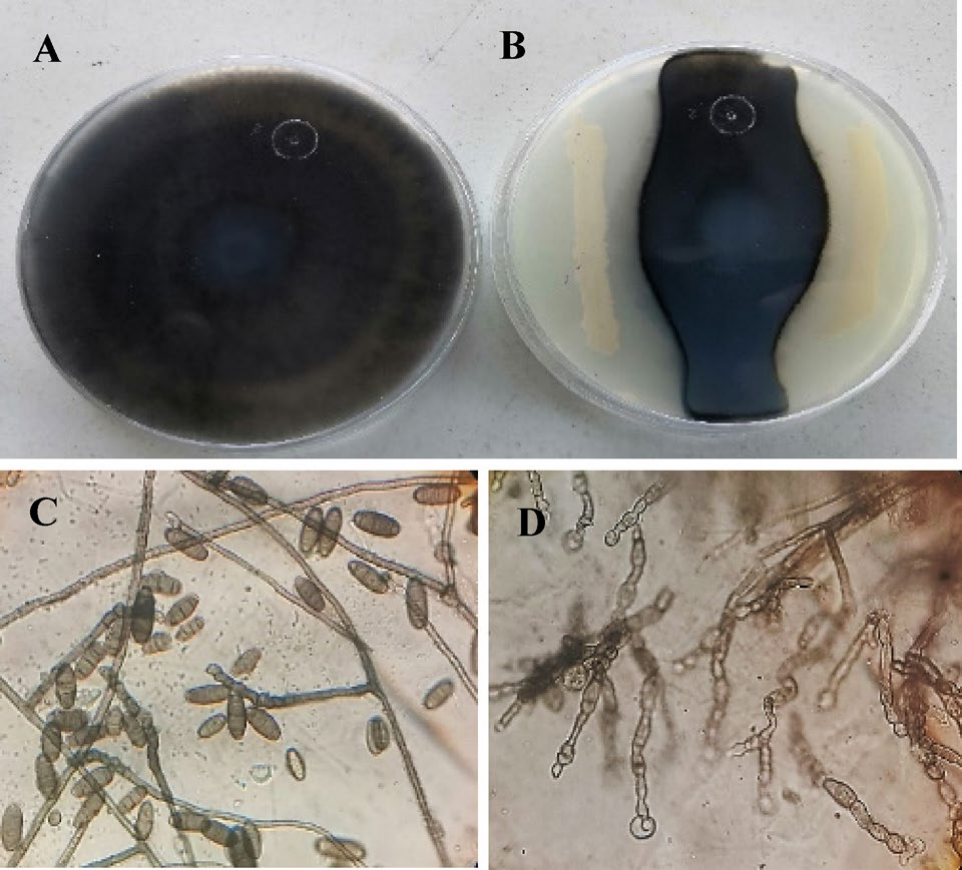
Morphological abnormalities in the mycelia of *Bipolaris sorokiniana* upon interaction with *Enterobacter cloacae* endophytic bacteria. Images (**A**,** C**) show the untreated (control) *Bipolaris sorokiniana* culture, mycelia and spores. Images (**B**,** D**) show inhibition of mycelium, swelling and segmentation and deformed hyphae.

The result also improved that *E. cloacae* strain RO2 caused significant morphological changes in *B. sorokiniana* mycelia under the light Microscope examination, primarily manifesting as inhibition of mycelium, swelling, segmentation and deformed hyphae (Figs. 6B and D). In contrast, untreated (control) *B. sorokiniana* culture were normal and well developed (Figs. 6A and C). Endophytic bacteria inhibit plant pathogenic fungi by producing antimicrobial metabolites that can destroy and lyse mycelia^89,62^. Recent researches have also shown that *B. sorokiniana* DNA was harmed by the *B. amyloliquefaciens* DB2 culture filtrate^68^. *B. velezensis* JK-1 culture filtrate possessed the capacity to destroy *B. sorokiniana’s* cell membrane integrity, leading to the accumulation of ROS^5^. In addition, Yi et al.^90^ reported that cyclic lipopeptides from *B. mojovensis* B1302 could affect the growth of *B. sorokiniana* and result in mycelial swelling and distortion.

**Fig. 7:**
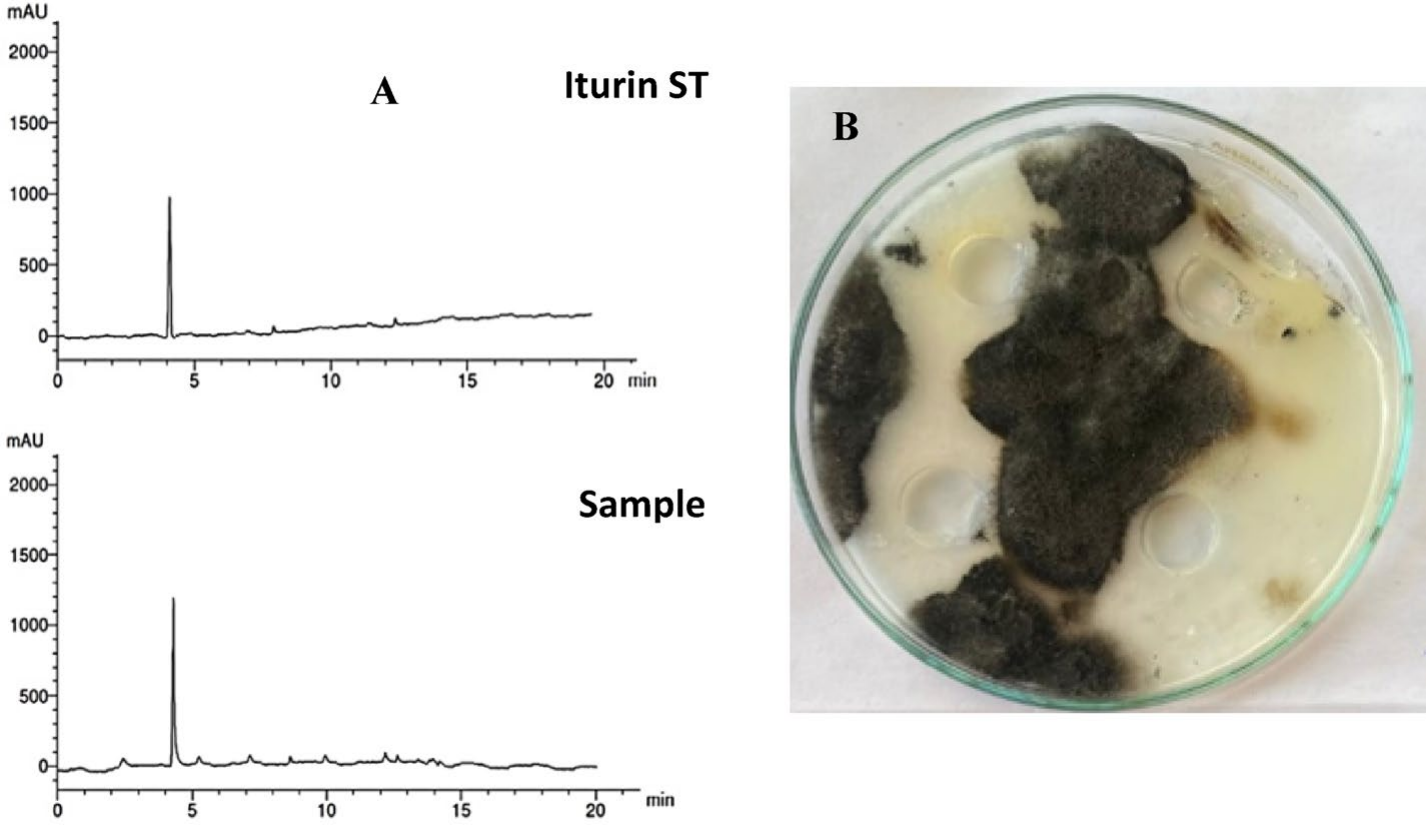
(**A**) HPLC analysis of lipopeptide extract from *Enterobacter cloacae* detected under UV at 240 nm. (**B**) Bioassay activity of the crude lipopeptide extract using well diffusion method (100 µL) against *Bipolaris sorokiniana* on PDA media.

*Bacillus*,* Enterobacter*, and *Pseudomonas* genera could be a source of bioactive metabolites with antimicrobial activities such as cyclic lipopeptides (CLP), including iturins, fengycins, and surfactins, which act against plant pathogenic fungi^91–93^. Our result of HPLC analysis of lipopeptide extract obtained from *E. cloacae* revealed the occurrence of lipopeptide like Iturin as shown in Fig. 7(A), which is an example of a family of cyclic antimicrobial peptides that inhibit the growth of fungi^94^. The crude extracts biological activity was investigated using a well diffusion method (100 µL) against *B. sorokiniana* on PDA (Fig. 7B**)**, which showed that *E. cloacae’*s extract had an inhibitory effect on *B. sorokiniana*. Our findings also align with the results of Mandal et al.^95^ that the HPLC of *Citrobacter* and *Enterobacter* strains purified antimicrobial lipopeptides, confirming the occurrence of numerous lipopeptide antibiotics such as iturin, surfactin and fengycin. Few researches describe the production of these lipopeptides by Gram-negative bacteria^95,96^. Jemil et al.^97^ reported that lipopeptides produced by *E. cloacae* C3 exhibited promising antifungal activity against *(A) niger* and *F. oxysporum.* Similar results are reported by Jadhav et al.^98^, who stated that biosurfactants produced by *Enterobacter* sp. MS16 showed a potential antifungal activity and prevented the growth of fungal spores. Mukherjee et al.^99^ reported that iturin A C-15 cyclic lipopeptide (SS1-3-P) was obtained from *E. cloacae* SS1-3, and it was purified and identified using reverse-phase HPLC. Yi et al.^90^ extracted CLP from *(B) mojovensis* B1302 and investigated its inhibitory effect on *B. sorokiniana* and other pathogenic fungi.

**Fig. 8:**
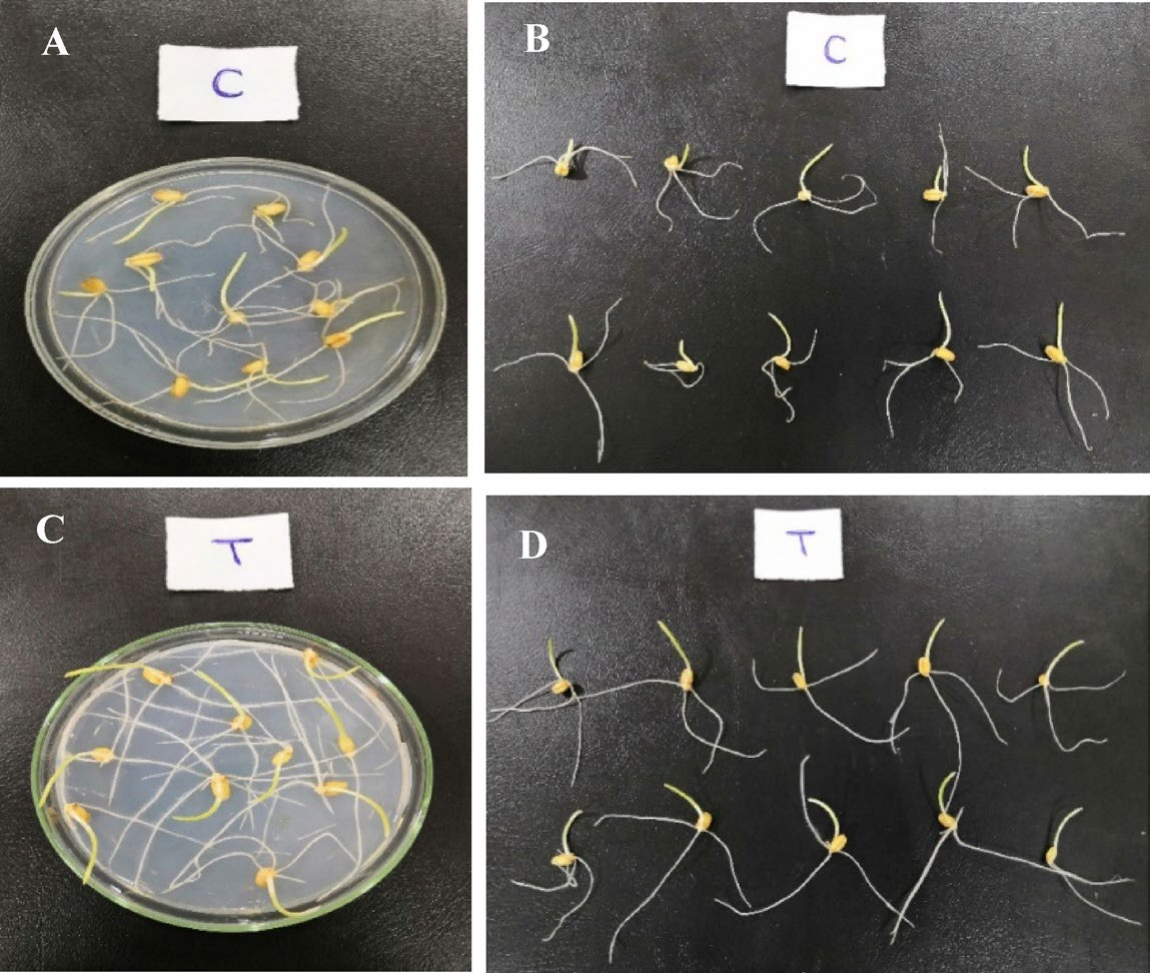
Seedling establishment and growth promotion analysis of *Enterobacter cloacae* using (*Triticum aestivum* L.) seeds. (**A**) and (**B**) showing seeds soaked in water as a control, (**C**) and (**D**) showing seeds soaked in a 10 mL cell-free filtrate of *E. cloacae*.

Bio-priming with PGPB is one of the cost-effective and environmentally friendly ways to boost seed growth in the early stages of development^100^. The use of beneficial PGPBs such as *Enterobacter* spp.^101^, *Pseudomonas* spp.^102^, and *Bacillus* spp.^103^, as bio-priming agent, has been utilized to improve plant growth under stress, seed germination and nutrient uptake. In general, the bio-inoculant exhibit a number of functions, such as the synthesis of plant growth hormones, such as auxins, gibberellins, cytokinins, and abscisic acid, as well as production of secondary metabolites^104^. The growth promotion potential of *E. cloacae* RO2 on wheat seeds was tested on the seedling stage, as shown in Fig. 8 and Table 3. The results showed that seed bio-priming with *E. cloacae* seeded on water agar media enhanced seedling growth and significantly improved shoot height, radicle length and seedling length as compared to the control (bio-primed with water). Shoot length was significantly higher in *E. cloacae*-primed seedlings compared to water-primed ones (t = -4.301, df = 4, *p* = 0.013). Also, seedling’s radicle length reached 4.85 ± 0.257 cm in *E. cloacae*-primed as compared to the control (3.60 ± 0.190 cm), and seedling fresh and dry weights are 1.173 ± 0.062 and 0.468 ± 0.025 in comparison with the control (1.105 ± 0.058 and 0.449 ± 0.024, respectively) (Fig. 8).

Our findings are in agreement with Khalifa et al.^86^, who demonstrated that *E. cloacae* MSR1 inoculation significantly improved the root dry weight and roots length of *Medicago sativa*. The MSR1-inoculated roots reached a length of 12.5 cm compared to the control (8 cm). Also, Suprapta et al.^105^ reported that *E. cloacae* considerably improved the growth parameters of rice seedlings. Bio- priming of seeds enhances the synthesis of proteins and DNA and assists in the development of mitochondria. Bio-priming, which involves soaking seeds in a bacterial suspension, stimulates bacterial colonization, triggers imbibition, and activates germination metabolic processes. Thus, this technique increases nutrient uptake, which benefits seed and plant health in general^106,107^. Similarly, the notable increase in growth parameter, along with the reduction of disease caused by *F. solani* was noticed in pea plants inoculated with *B. subtilis* and *B. halotolerans*, which produced the lipopeptides surfactin, fengycin, and bacillomicin, in addition to IAA, protease, cellulase, glucanase, as well as siderophores^108^.

## Conclusion

The present study demonstrated that the endophytic *Enterobacter cloacae* isolated from *Portulaca oleracea* L. exhibits remarkable biocontrol potential against *Bipolaris sorokiniana*, and has PGP traits. The isolate effectively inhibited the mycelial growth of the pathogen through multiple mechanisms, including the production of siderophores, hydrolytic enzymes, and antifungal metabolites. In addition, *E. cloacae* showed significant PGP attributes, such as phosphate solubilization, and N_2_ fixation, which collectively contribute to improved nutrient acquisition and enhanced plant vigor. The dual functionality of *E. cloacae* simultaneously suppressing *B. sorokiniana* *in vitro* and stimulating wheat seedling growth highlights its potential as a biocontrol agent with plant growth promoting traits. These findings underscore the importance of exploring plant-associated endophytic bacteria as eco-friendly tools for integrated disease management and crop productivity improvement. Although the study’s findings were positive, many limitations were found, including “iturin-like lipopeptide” which was evaluated only by comparison of the Iturin standard using HPLC retention time, we acknowledge that the identification is putative, not confirmed, and that detecting biosynthetic genes will need to be done in our future work. We emphasize that many biocontrol and sustainable disease management studies initially rely on *in vitro* assays to establish efficacy before progressing to field-scale validation. Therefore, our study represents a critical first step. Field or greenhouse investigations are warranted to validate its efficacy under diverse environmental conditions. We acknowledge that our findings demonstrate preliminary antifungal potential under controlled laboratory conditions.

## Data Availability

Sequence data that support the findings of this study have been deposited in the NCBI [https://www.ncbi.nlm.nih.gov/nuccore/PV739238].

## Abbreviations

CMC: Carboxymethylcellulose
CAS: Chrome Azurol S
DMRT: Duncan’s multiple range test
IAA: Indole-3-acetic acid
PGPB: Plant growth-promoting bacteria
PDA: Potato dextrose agar
ROS: Reactive oxygen species
RP-HPLC: Reversed-phase high-performance liquid chromatography
VOCs: Volatile organic compounds
